# Extramedullary relapse of IgA-lambda myeloma after recent bortezomib therapy: a case report

**DOI:** 10.4076/1757-1626-2-7456

**Published:** 2009-09-14

**Authors:** Deirdre F Waterhouse, Geraldine A Moloney, Fatma S Gargoum, Peter S Hayden, Tom O’Gorman

**Affiliations:** 1 Department of Gastroenterology, University College HospitalGalwayIreland; 2 Department of Haematology, University College HospitalGalwayIreland

## Abstract

Intracranial plasmacytomas are an uncommon presentation of extramedullary relapse of multiple myeloma. The optimal management of extramedullary plasmacytomas remains unclear, with initial reports of bortezomib showing promising clinical results. We describe a case of multiple extracellular, including intracranial, plasmacytoma, with no evidence of marrow involvement, in a patient with relapsed IgA multiple myeloma. To our knowledge, this is the first reported case of a patient with rapid extramedullary relapse of disease despite recent exposure to bortezomib and dexamethasone.

## Case presentation

A 64 year old female patient consulted her ophthalmologist because of onset of blurred vision. This diplopia was worse on looking straight ahead, and to the left. Initially, intermittent, the diplopia became constant, at which point she attended her ophthalmologist. She denied any other symptom.

Physical examination revealed a primary position esotropia, binocular horizontal diplopia and a horizontal gaze-evoked nystagmus. These findings were consistent with a left sixth cranial nerve palsy. Notably, additional signs of increased ICP (e.g. papilloedema) were absent and the remaining neurological examination was unremarkable.

This lady was diagnosed with IgA Multiple Myeloma (MM) in 2004, for which she commenced treatment with thalidomide and dexamethasone. This was discontinued after three cycles, due to proximal myopathy. She then received melphalan and prednisolone as second-line treatment, with suppression of serum monoclonal band. An elective autologous stem cell transplant (ASCT), with cyclophosphamide pre-conditioning, was performed in 2005. A subsequent disease relapse two years later was initially treated with melphalan and prednisolone, but this regimen was complicated by pancytopaenia. At this point, bortezomib and dexamethasone therapy was commenced, with an excellent response [1,2]. Bortezomib was administered as eight 3-week treatment cycles of bortezomib (1.3 mg/m^2^ as a single intravenous bolus on days 1, 4, 8, 11) followed by a 10-day rest period (days 12 through 21). In addition, the patient received dexamethasone 20 mg orally once daily on the day of bortezomib injection and the day thereafter (days 1, 2, 4, 5, 8, 9, 11, and 12). An SPEP on completion of this chemotherapy, one month prior to this presentation, demonstrated complete suppression of serum paraprotein, although B_2_ microglobulin was slowly rising (2.66 mg/L).

**Figure 1a and b.**
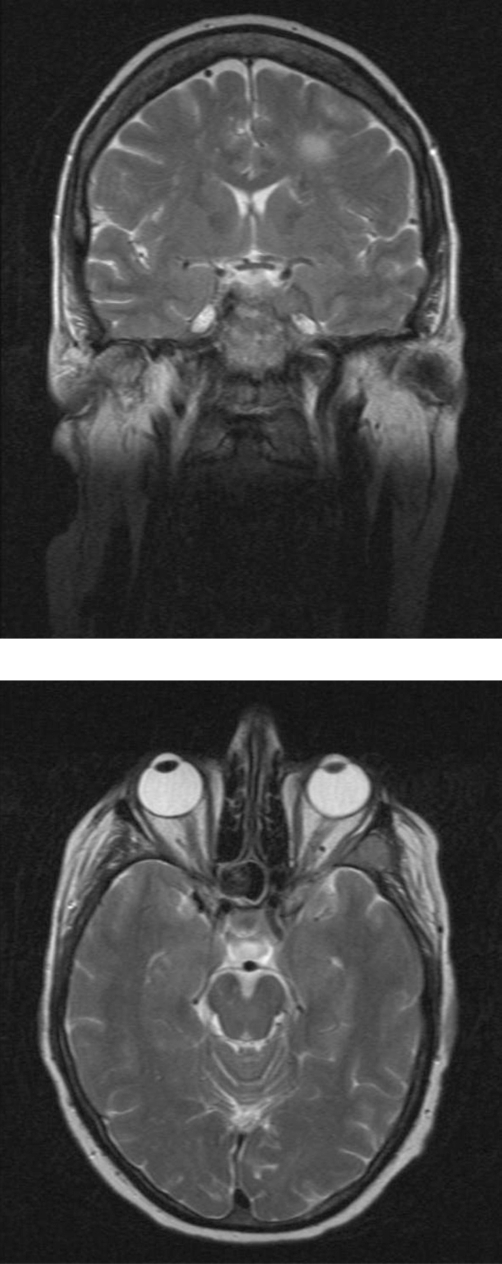
Magnetic resonance imaging of patient’s brain shortly after admission. Gadolinium enhanced Magnetic Resonance Image demonstrating two enhancing meningeal-based lesions one in the left frontal extra-axial region, and the second lesion on the left side of the cavernous sinus. The lesion in the left cavernous sinus is also associated with bone destruction. Given this patients past medical history, these images are consistent with extra axial soft tissue deposits of myeloma.

**Figure 2.**
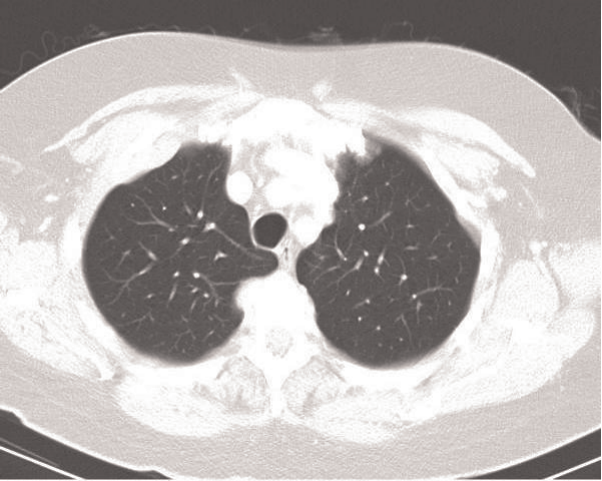
Computerized tomographic imaging of patient's thorax at presentation. A computerized tomogram of the patient's thorax performed soon after admission demonstrating multiple paravertebral and pleural based masses with epidural extension.

On admission, MRI brain demonstrated enhancing meningeal-based lesions in the left frontal region and the left side of the cavernous sinus (Figure 1a and b). Immunofixation electrophoresis demonstrated an IgA-lambda monoclonal band, with free lambda chain measuring 142 mg/dl. Subsequent CT TAP demonstrated multiple pelvic osseous lytic lesions as well as paravertebral and pleural based masses (Figure 2). Notably, there was no increase of plasma cells in bone marrow.

A diagnosis of extramedullary relapsed MM (paravertebral, pleural and intracranial masses) after recent bortezomib therapy was made.

## Discussion

We describe a case of multiple extracellular plasmacytoma, with no evidence of marrow involvement, in a patient with relapsed IgA MM. To our knowledge, this is the first reported case of a patient with rapid extramedullary relapse of disease despite recent exposure to bortezomib and dexamethasone.

While rare, extramedullary relapse of MM following ASCT has previously been reported, with a rate of relapse of 14% in the Spanish Registry of Transplants.[3] When relapse does occur, prognosis is poor, with mortality of 73%, and median survival of 12 months [4]. Intracranial plasmacytomas are rare, with the Arkansas group demonstrated an incidence of CNS involvement in 1% of relapse after ASCT [5]. Intracranial plasmacytomas without medullary relapse are even rarer, with only a few reported cases in the literature [6,7].

The optimal management of extramedullary plasmacytomas remains unclear. A 2004 review by the UK Myeloma Forum of treatment modalities for plasmacytoma, either solitary bone or extramedullary, concluded that while the available evidence was limited, localised radiotherapy was the favoured treatment option [6]. In recent times, localised radiotherapy has been largely superseded by bortezomib, a proteasome inhibitor of NF-KB, which is now the treatment of choice for extramedullary disease [8]. Initial reports of bortezomib for extramedullary MM have shown promising clinical results [5,9,10].

Our patient had recently completed a course of bortezomib, and despite this, had a rapid presentation with extramedullary disease. This relapse may suggest a high degree of chemo-resistance in our patient, a hypothesis supported by several case reports that propose a high degree of chemo-resistance in CNS and skull lesions that precede medullary relapse [7]. Additionally, this lady had both paravertebral and pleural plasmacytomas. Spinal and surrounding tissue have been identified as sites of relapse in many series, leading to the theory of so-called “sanctuary sites”. Terpos et al have proposed that pre-conditioning with high dose chemotherapy may permit the escape, and subclinical seeding, of an extramedullary clone of plasma cells with extensive chemoresistance [2].

The clinical phenomenon of an extramedullary “escape” has previously been recognised with thalidomide regimes. Anagnostopoulos et al. described the occurrence of a ‘hyposecretory’ progression in 48/103 patients, with reduction of serum paraprotein levels, despite extensive plasmacytosis of bone marrow. Bone marrow progression occurred with and without concomitant extramedullary involvement. This discordance was also identified in two patients with soft tissue plasmacytomas, and one patient each with cervical and mediastinal lymph node plasmacytomas. As the anti-myeloma mechanisms of thalidomide are not fully elucidated, they have suggested that this treatment allows selection of a more immature clone, and thus reduced levels of paraprotein [11]. Experience with thalidomide in the treatment of extramedullary disease has been disappointing, with suggestions that the stroma of bone marrow are necessary for its inhibition of plasma cells [8].

## Conclusion

Multiple plasmacytomas, including intracranial involvement, is an uncommon presentation of extramedullary relapse of MM. Although promising in treating extensive plasmacytosis of bone marrow, this patient’s clinical course suggests that bortezomib may not be effective for treating all extramedullary relapses, or a highly chemo-resistant clone (as suggested by her extensive prior treatments).

## Abbreviations

ASCT: autologous stem cell transplant
CNS: central nervous system
ICP: intracranial pressure
MM: multiple myeloma
MRI: magnetic resonance imaging
SPEP: serum protein electrophoresis
TAP: total alkaline phosphatase
